# Tissue-dependent transcriptional and bacterial associations in primary sclerosing cholangitis-associated inflammatory bowel disease

**DOI:** 10.12688/wellcomeopenres.16901.2

**Published:** 2022-11-04

**Authors:** Nicholas E. Ilott, Mastura Neyazi, Oxford Translational Gastroenterology Unit Investigators, Carolina V. Arancibia-Cárcamo, Fiona Powrie, Alessandra Geremia

**Affiliations:** 1 The Kennedy Institute of Rheumatology, Nuffield Department of Orthopaedics, Rheumatology and Musculoskeletal Sciences, University of Oxford, Oxford, OX3 7FY, UK; 2 Translational Gastroenterology Unit, Nuffield Department of Clinical Medicine, Experimental Medicine Division, and NIHR Oxford Biomedical Research Centre, Oxford University Hospitals NHS Foundation Trust, John Radcliffe Hospital, University of Oxford, Oxford, OX3 9DU, UK

**Keywords:** Primary sclerosing cholangitis, IBD, transcriptomics, microbiome

## Abstract

**Background: **Patients with primary sclerosing cholangitis (PSC) frequently have co-ocurring ulcerative colitis (UC) and develop colorectal cancer. Colorectal cancer risk in patients with PSC-associated ulcerative colitis (PSC/UC) is elevated relative to patients with ulcerative colitis (UC) alone, reasons for which remain obscure. Understanding the molecular and microbial basis for differences between these two patient groups and how these vary across intestinal sites is important for the development of therapies to prevent colorectal cancer development in at-risk individuals.

**Methods: **We employed ribonucleic acid sequencing (RNA-seq) analysis of biopsy samples across three intestinal tissue locations (ileum, caecum and rectum) in patients with PSC/UC (ileum n = 7, caecum n = 7, rectum n = 7), UC (ileum n = 9, caecum n = 10, rectum n = 10) and healthy controls (ileum n = 11, caecum n = 9, rectum n = 12) to determine tissue-dependent transcriptional alterations in PSC/UC. We also performed 16S ribosomal RNA (rRNA) amplicon sequencing to determine bacterial associations with PSC/UC.

**Results: **Tissue-defining transcriptional signatures revealed that the ileum was enriched for genes involved in lipid and drug metabolism, the caecum for activated immune cells and the rectum for enteric neurogenesis. Transcriptional alterations relative to healthy control samples were largely shared between patients with PSC/UC or UC although were distinct across tissue locations. Nevertheless, we observed reduced expression of gamma-glutamyl transferase 1 ( *GGT1*) specifically in the ileum and caecum of patients with PSC/UC.

Analysis of the bacterial component of the microbiome revealed high inter-individual variability of microbiome composition and little evidence for tissue-dependency. We observed a reduction in *Parabacteroides* relative abundance in the rectum of patients with PSC/UC.

**Conclusions: **The role of gamma-glutamyl transferase in maintaining the redox environment through the glutathione salvage pathway makes our observed alterations a potential pathway to PSC-associated colorectal cancer.

## Introduction

Primary sclerosing cholangitis (PSC) is a chronic progressive disorder of the hepatobiliary system characterised by inflammation, fibrosis, and stricturing of the intrahepatic and extrahepatic bile ducts. A common co-occurring disease in patients with PSC is inflammatory bowel disease (IBD), with a prevalence of 60–80%. While most frequently diagnosed as ulcerative colitis (UC) ^1^, clinical features of colitis in patients with PSC are somewhat distinct. These features include more frequent pancolitis or right-sided patchy inflammation, rectal sparing, “backwash ileitis” and quiescent course ^2^. Further, an important differential disease course between patients with PSC/UC and patients with UC is the fivefold increased risk of colorectal cancer in PSC/UC relative to UC ^3^. The reasons for this increased risk are currently unclear but in the context of colitis-associated cancer may involve pro-inflammatory immune phenotypes that promote cell growth and proliferation ^4^, reduced ability to clear deoxyribonucleic acid (DNA)-damaging reactive oxygen species ^5^, defective DNA-damage repair mechanisms or alterations to the gut microbiota that favour genotoxic species ^6, 7^.

Clinical and disease course distinctions are important when considering a molecular and cellular basis for disease. Indeed, as a complex disease, genome-wide association studies (GWAS) have identified multiple genetic variants of small effect associated with PSC. The most highly associated risk variants are those that reside in the human leukocyte antigen (HLA) locus with non-HLA variants predominantly associated with adaptive immunity ^3^. Nevertheless, the genetic correlation between PSC/UC and UC is relatively low ^3^, suggesting distinct genetic architectures between these diseases. Furthermore, while immune phenotypes that differentiate PSC/UC from UC have not been extensively studied, and although broadly similar in major cell types ^8^, there is some evidence to suggest an increased frequency of innate lymphoid cells and interferon gamma (IFN-γ) producing CD4 T cells in the colon of patients with PSC/UC relative to UC ^9^. RNA-sequencing analysis of colonic biopsies has provided links between PSC/UC and increased expression of genes involved in bile acid homeostasis and decreased expression of genes involved in DNA damage repair relative to patients with UC ^8^. These data support alterations in liver-gut cross-talk that distinguishes colonic transcriptomes of patients with PSC/UC from those with UC.

The aforementioned cellular and molecular studies in PSC/UC have focussed on colonic samples. However, observed variation in tissue involvement between PSC/UC and UC provides an impetus to extend studies to both the large and the small intestine that may have distinct roles to play in pathogenesis. Indeed, the structure and function of the intestine varies across its length. The small intestine is home to a high density of absorptive enterocytes that promote nutrient uptake where the main role of the colon is to remove water from stool and accommodate a high density of microorganisms – the microbiome - that aid in digestion, peristalsis and immune function. Subtle and significant differences in cellular composition between small intestinal and colonic sites in terms of epithelial ^10^ and immune cell types ^11^ supports a requirement to study isolated and integrated effects on disease across tissue sites.

There is growing evidence for a role of the gut microbiome in PSC pathogenesis. Indeed, multiple microbiome association studies in both stool ^12– 16^ and the intestinal mucosa ^8, 17, 18^ have identified alterations in the bacterial component of the microbiome in PSC and/or PSC/UC. Increased relative abundance of *Enterococcus, Veillonella* and *Streptococcus* genera have been reported in multiple studies ^19^, with *Klebsiella pneumoniae*-harbouring microbiomes from patients with PSC/UC being implicated as a driver of Th17 responses in gnotobiotic mouse models ^20^. Nevertheless, more work is required to understand mechanistic links between disease processes and members of the gut microbiome.

In this study we have expanded on existing knowledge of the host transcriptome and mucosa-associated microbiome in PSC/UC across multiple relevant intestinal sites. Using RNA-seq we have explored transcriptomes across the terminal ileum, caecum and rectum, providing an in-depth assessment of tissue-defining gene expression signatures and how these signatures vary in patients with PSC/UC and UC relative to healthy controls. In addition to host transcriptomics, we have explored the mucosa-associated bacterial component of the microbiome using 16S ribosomal ribonucleic acid (rRNA) amplicon sequencing across the same intestinal sites, revealing modest associations in disease. 

## Methods

### Participant recruitment

All study participants were recruited from the department of Gastroenterology, University of Oxford, and samples were collected as part of the Translational Gastroenterology Unit (TGU) biobank in the John Radcliffe Hospital in Oxford and part of the BRC Oxford IBD Cohort (REC 09/H1204/30) and BRC GI Illness Biobank (REC 16/YH/0247). Participants with a previous formal diagnosis of PSC/UC or UC according to clinical, radiological, endoscopic and histological criteria were identified from our databases and approached at outpatient clinic or endoscopy appointments to confirm consent and obtain intestinal samples. There were no specific inclusion/exclusion criteria. The sample size was based on what was achievable in the timeframe of the project.

For RNA-seq analysis, samples were collected from 13 control participants, 9 patients with PSC/UC and 10 patients with UC. Controls consisted of patients undergoing colonoscopy for chronic diarrhoea/abdominal pain, iron-deficiency anaemia, or follow-up after previous polyps, with findings of uninflamed intestine. Biopsies were taken from macroscopically and microscopically - as confirmed by an independent pathologist - normal colon and terminal ileum. For microbiome profiling, samples were collected from 16 control participants, 20 patients with PSC/UC and 18 patients with UC. 

### Ethical considerations

All patients provided written informed consent for participation in this study through the TGU Biobank (BRC Oxford IBD Cohort (University of Sheffield Research Ethics Committee (REC) 09/H1204/30) and BRC GI Illness Biobank (University of Sheffield REC 16/YH/0247).

### Sampling intestinal sites through biopsy

Biopsies were obtained from ileum, caecum and rectum from patients with PSC/UC and patients with UC alone, undergoing colonoscopy for surveillance or disease activity and extension assessment, and non-inflammatory controls undergoing colonoscopy for colorectal cancer screening or chronic abdominal pain/diarrhoea with a macroscopically and microscopically normal colon. Endoscopic activity of PSC/UC and UC was defined by the Ulcerative Colitis Endoscopic Index of Severity (UCEIS) score ^21, 22^ and microscopic inflammation by the Nancy score ^23^. Other clinical and demographic information, including current medications, were obtained from our clinical patient database.

### Nucleic acid extraction

Biopsies were collected in RNALater ^TM^ TissueProtect (Qiagen) and stored according to manufacturer guidelines. On the day of DNA/RNA extraction, samples were thawed at room temperature, RNALater was removed and 350–700ul of RLT Buffer (Qiagen) with 10ul β-mercaptoethanol (Sigma) was added to each sample and samples were transferred to Soft Tissue Homogenizing CK14 Kit vials (Stretton Scientific). Samples were processed using Precellys 24 (Bertin Instruments) homogeniser (40s at 6500 RPM). 350uL of 70% ethanol was added to homogenised tissue lysates and lysates were transferred to QIAshredder column (Qiagen cat 79656) and subsequently transferred to the AllPrep DNA/RNA MicroKit (Qiagen). An on-column DNase digestion was performed for each sample using the RNAse Free DNAse Kit (Qiagen). Samples were eluted from columns in 30uL RNAse free; DNAse free H _2_O and RNA concentrations and 260/280 ratios were determined on a NanoDrop 1000 (Thermo Fisher).

### RNA-seq

Selection of PolyA+ mRNA, generation of double stranded cDNA and library construction were performed using NEBNext Poly(A) mRNA Magnetic Isolation Module (E7490) and NEBNext Ultra II Directional RNA Library Prep Kit for Illumina (E7760L) with Illumina adapters and barcode tags (dual indexing, based on 24). The RNA-seq libraries were pooled and sequenced on an Illumina NovaSeq6000 as 150bp paired end reads. This generated an average of 71.7M (324.5K – 104.5M) paired reads per sample. We removed one sample from downstream analysis based on markedly low read counts (324.5K). The remaining samples had an average of 72.4M (27.5M – 104.5M) paired reads.

### 16S rRNA amplicon sequencing

For bacterial 16S rRNA gene amplicon sequencing, the variable V3 and V4 regions of the 16S rRNA gene were amplified from genomic DNA using the primers (standard IUPAC nucleotide nomenclature): Forward Primer = 5' TCGTCGGCAGCGTCAGATGTGTATAAGAGACAGCCTACGGGNGGCWGCAGTCGTCGGCAGCGTCAGATGTGTATAAGAGACAGCCTACGGGNGGCWGCAG, Reverse Primer = 5’ GTCTCGTGGGCTCGGAGATGTGTATAAGAGACAGGACTACHVGGGTATCTAATCC

The amplicons were then attached with indices and Illumina sequencing adapters using the Nextera XT index kit. The 16S amplicon libraries were pooled and sequenced in an Illumina MiSeq v3 flowcell as 300 paired end reads. Samples were removed from downstream analysis if they had fewer than 10000 paired reads (n = 19).

### Processing RNA-seq data

Raw reads were assessed for quality using Fastqc (v0.11.7). Paired reads were aligned to the human genome (build hg38) using HISAT2 ^25, 26^ (v2.1.0). Gene counts were produced using featureCounts ^27^ (v1.6.0) against Ensembl genesets (Ensembl build 91 ^28^). Initial principal components analysis of transformed read counts (log2 counts per million) across tissues revealed three outlying patient samples. The patterns of gene expression were indicative of a sample swap and these samples were removed from further analysis. The resulting dataset therefore consisted of PSC/UC (ileum n = 7, caecum n =7, rectum n = 7), UC (ileum n = 9, caecum n = 10, rectum n = 10) and healthy controls (ileum n = 11, caecum n = 9, rectum n = 12).

### Generating tissue-defining transcriptional signatures

In all analyses of RNA-seq data, genes that had ≥ 10 reads in ≥ 7 samples (minimum number of samples for any tissue-disease group) were taken forward. We used the likelihood ratio test (LRT) in DESeq2 ^29^ to determine transcriptional differences among tissue locations comparing ileum, caecum and rectum samples from healthy individuals in a single test. Given that all participants contributed samples from each tissue location, the effect of individual variation was controlled for in the analysis (i.e. the DESeq2 design was specified as ~Individual + tissue). Genes were considered as significantly varying by tissue at an adjusted p-value < 0.05. Significantly varying genes were visualised in a heatmap and clustered using Manhattan distance and Ward.D clustering ( *pheatmap*, R3.6.1). Genes were assigned to co-varying clusters by passing an *hclust* object (Manhattan distance and Ward.D clustering) to the *dynamicTreeCut* package ^30^. Using *method="hybrid"* and *cutHeight=30000* as parameters to *dynamicTreeCut* led to the identification of five clusters of genes.

### Gene ontology pathway enrichment analysis

Pathway enrichment in tissue-defining gene clusters was performed using the hypergeometric test implemented in runGO.py from cgat-apps ^31^. Genes from each cluster were output as the foreground datasets with all genes that had been assigned to a cluster used as the background set. The Gene Ontology (GO) biological pathway gene sets available from the Molecular Signatures Database (MSigDB, c5.bp.v6.1) was formatted using gmt2tsv.py to make it compatible with runGO.py. Gene sets were considered enriched at a Benjamini-Hochberg adjusted p-value < 0.05.

### Differential expression across disease groups

We performed differential expression analysis in a pairwise manner (i.e. PSC/UC vs healthy, UC vs. healthy and PSC/UC vs. UC) in each tissue location separately, using DESeq2 ^29^. Genes were considered significantly differentially expressed at an adjusted p-value < 0.05. Results from each of these comparisons were compared to each other using a combination of Venn diagrams ( VennDiagram, R3.6.1) and scatterplots of log2(fold changes).

### Enrichment of tissue-defining clusters among disease-associated gene sets

We used our previously defined tissue-defining gene clusters as input gene sets into runGO.py. Foreground gene sets were those genes that were called as differentially expressed in the pairwise differential expression analyses across diseases. The background gene set was any gene that had been assigned to a cluster. Clusters enriched in differential gene sets were considered significant at a Benjamini-Hochberg adjusted p-value < 0.05.

### Analysis of gamma-glutamyl transferase in intestinal single-cell RNA-seq data

Single-cell transcriptomic data ^10^ with accession number GSE125970 were downloaded from the gene expression omnibus (GEO). Processed data that represented unique molecular identifier (UMI) counts were used for the analyses (GSE125970_raw_UMIcounts.txt). Therefore annotated cell clusters that had been previously described ^10^ were used to determine epithelial cell types that expressed *GGT1*. Any cell with at least one UMI annotated to *GGT1* was considered *GGT1* positive (GGT1+).

### Cell deconvolution analysis

To predict changes in cell composition between groups we used CIBERSORTx ^32^ for cell deconvolution. We utilised single cell RNA-seq data of immune cells from the human gut ^33^ as the input signatures matrix to CIBERSORTx. This gave us the capacity to predict cellular fractions of 25 immune cell types (IgA plasma B cell, memory B cell, CD8 T cell, gamma-delta T cell, mast cell, innate lymphoid cell, macrophage, natural killer cell, follicular B cell, IgG plasma B cell, Central memory T cell, cycling B cell, Treg, LYVE1 macrophage, Th1 cell, Th17 cell, conventional dendritic cell 1, conventional dendritic cell 2, cycling gamma-delta T cell, monocyte, activated CD4 T cell, T follicular helper cell, lymphoid dendritic cell, peripheral dentritic cell and cycling dendritic cell). Differential frequency of each cell type between either PSC/UC vs. healthy or UC vs. healthy was determined using a Wilcoxon rank sum test in R3.6.1. Significant differences were determined at a Benjamini-Hochberg adjusted p-value < 0.05.

### Processing 16S rRNA amplicon sequencing

Raw reads were assessed for quality using Fastqc (v0.11.7). Given a minimal overlapping region between paired reads, we continued analysis using the first read of the pair (V3 variable region). Reads were processed using DADA2 ^34^, implemented using a CGAT-core ^35^ based pipeline (pipeline_dada2.py) available at https://github.com/OxfordCMS/OCMS_16S. First, we trimmed reads to 200bp and removed primer sequences (17bp). The resulting 183bp reads were aligned against the human genome (hg38) using bowtie2 ^36^ (v2.3.4.1) and any aligned reads were filtered out. Alignment and filtering were performed using a CGAT-core based pipeline (pipeline_filter.py) available at GitHub. Samples that had fewer than 5 000 reads were removed from downstream analysis. The distribution of samples across tissue sites that were taken forward for analysis is shown in Table 1. Filtered reads were then taken through the DADA2 workflow of learning error rates, sample inference and taxonomy assignment. Taxonomy was assigned using the RefSeq-RDP16S_v2_May2018 database. Downstream analysis was performed in amplicon sequence variant (ASV) counts summed at the level of genus.

**Table 1.** Patient and sample characteristics. Quantitative variables are expressed as median (standard deviation) and categorical variables as number of samples. Numbers are broken down by tissue site as not all patients had a sample for every tissue site.

RNA-seq samples									
	Control			PSC/UC			UC		
	Ileum	Caecum	rectum	Ileum	Caecum	Rectum	Ileum	Caecum	Rectum
Total N	11	9	12	7	7	7	9	10	10
***Demographics***									
Age (Years)	46 (14.18)	42 (15.17)	49 (17.00)	59 (20.58)	60 (20.66)	59 (23.39)	54 (10.42)	53.5 (10.38)	53.5 (10.83)
Sex (F/M)	2/9	1/8	3/9	3/4	3/4	2/5	4/5	5/5	6/4
***Inflammation***									
UCEIS	NA	NA	NA	0 (0.76)	0 (0)	0 (0.76)	0 (1.92)	0 (1.81)	0 (1.73)
NANCY	NA	NA	NA	0 (0)	0 (0)	0 (0.38)	2 (1.41)	1 (1.40)	0 (1.41)
***Medication***									
URSO	0	0	0	7	7	7	9	9	9
Vedolizumab	0	0	0	0	0	0	0	0	0
ASA	0	0	0	7	7	7	7	8	8
Azathioprine	0	0	0	1	1	2	3	4	4
Steroids	0	0	1	0	0	1	0	0	0
***Liver function***									
Bilirubin (nMol/L)	12 (3.56)	9.5 (3.56)	12 (3.56)	14 (9.19)	14 (13.48)	14 (9.76)	6 (5.74)	7 (6.43)	7 (6.43)
ALP (IU/L)	86 (26.87)	86 (30.72)	86 (26.87)	97 (107.55)	97 (348.25)	94 (117.66)	57.5 (7.96)	57 (9.61)	57 (9.61)
ALT (IU/L)	22 (11.92)	28 (10.90)	22 (11.92)	24 (33.50)	24 (23.38)	19.5 (36.69)	27.5 (7.79)	30 (8.19)	30 (8.19)
16S amplicon sequencing samples									
	Control			PSC/UC			UC		
	Ileum	Caecum	rectum	Ileum	Caecum	Rectum	Ileum	Caecum	Rectum
Total N	14	14	13	19	16	18	16	18	14
***Demographics***									
Age (Years)	45 (16.29)	45 (16.90)	57 (19.37)	42 (18.87)	42 (19.05)	42 (19.71)	51.5 (12.16)	53.5 (12.48)	53.5 (12.90)
Sex (F/M)	5/9	5/9	4/9	8/11	8/8	7/11	7/9	8/10	/7
***Inflammation***									
UCEIS	0 (0)	0 (0)	0 (0)	0 (1.46)	0 (1.30)	0 (1.52)	0 (1.93)	0 (1.87)	0 (0.94)
NANCY	0 (0)	0 (0)	0 (0)	0 (1.02)	0 (0.89)	0 (0.86)	2 (1.45)	1 (1.45)	0 (1.12)
***Medication***									
URSO	0	0	0	15	12	14	0	0	0
Vedolizumab	0	0	0	1	1	0	0	0	0
ASA	0	0	0	18	15	17	13	15	11
Azathioprine	0	0	0	4	3	4	7	7	6
Steroids	0	1	2	2	2	2	0	0	0
***Liver function***									
Bilirubin (nMol/L)	10 (4.24)	7 (4.21)	8.5 (3.66)	9 (11.14)	10 (11.43)	12 (11.00)	7.5 (3.83)	8 (3.67)	9 (3.69)
ALP (IU/L)	82 (23.03)	83 (25.48)	81.5 (21.61)	97 (225.06)	94 (244.88)	94 (235.07)	61 (18.41)	61 (17.50)	59.5 (17.79)
ALT (IU/L)	23 (8.91)	22 (9.51)	21.5 (9.51)	31 (62.79)	31.5 (67.12)	30 (44.63)	27.5 (8.40)	26 (8.01)	25 (9.49)

UCEIS = Ulcerative Colitis Endoscopic Index of Severity. Nancy = Nancy histologic index. URSO = Ursodeoxycholic acid. ASA = 5-aminosalicylates. ALP = Alkaline Phosphatase. ALT = Alanine aminotransferase.

### Analysis of 16S rRNA amplicon sequencing controls

To aid interpretation of our 16S rRNA amplicon sequencing data we included a series of controls in the experiment. First, we utilised a mouse model (C57BL/6) with a defined microbial community with 12 members ( *Acutalibacter muris* KB18, *Akkermansia muciniphila* YL44, *Bacteroides caecimuris* 148, *Bifidobacterium animalis* YL2, *Blautia coccoides* YL58, *Clostridium clostridioforme* YL32, *Clostridium inocuum* 146, *Enterococcus faecalis* KB1, *Flavonifractor plautii* YL31, *Lactobacillus reuteri* 149, *Muribaculum intestinale* YL27 and *Turicimonas muris* YL45) ^37^. All experiments were conducted in accordance with the UK Scientific Procedures Act (1986) under a Project License (PPL) authorized by the UK Home Office (P508FFA1F). Animals were housed in accredited animal facilities at the University of Oxford and provided with sterile water and food *ad libitum*, and environmental enrichment. This section of the manuscript is reported in line with the Animal Research: Reporting of In Vivo Experiments (ARRIVE) guidelines ^38^. Germ free C57BL/6J mice were stably colonized with a defined consortium of 12 bacterial members of murine intestinal flora to generate a sDMDMm2 (MM12) colony that was maintained in sterile flexible film isolators.

We serially diluted (1:10 series) caecal contents from a single mouse from this colony (1–1:1000000) reasoning that reagent contaminants would show up in the more dilute samples as has been previously described in single colony experiments ^39^. All samples that were extracted were included in the analysis and no criteria were set for exclusion. The least dilute samples acted as a pseudo-positive control i.e. we could determine whether we were able to sequence members of the community but not accuracy in relative abundance estimations (we did not know this beforehand). Any ASVs that were assigned to a genus that was not part of this experimental community was flagged as a potential contaminant. In addition to these controls we also sequenced a negative control sample. This was extracted DNA from an endoscopic brush that had been passed through the endoscope during colonoscopy, once the caecum was reached but had not been used to brush the mucosa. Any ASV found in this endoscope control sample was added to the list of potential contaminants for future cross-referencing.

### Exploratory analysis of 16S rRNA amplicon data

We performed alpha diversity analysis using the phyloseq ^40^ package (R3.6.1) and significance of disease group differences was determined using the Kruskall-Wallis test (Kruskal.test in R3.6.1). We explored beta-diversity associations with variables of interest (tissue location, experimental group) and covariates (sequencing batch and DNA extraction kit) using principal components analysis (PCA) of log10-transformed genus relative abundance estimates (prcomp in R3.6.1). We defined significant associations as a p-value < 0.05 in a univariate Adonis test (vegan package in R3.6.1) run for each variable separately. Given that we observed a significant effect (p < 0.05) of sequencing batch, we included this as a covariate in Adonis testing of both tissue location and disease. To determine the relationship between inter-individual and inter-tissue variability we performed dissimilarity analysis (Bray-Curtis dissimilarity implemented in phyloseq) both within-tissue sites (i.e. between individuals) and within individuals (i.e. between tissue-locations). Significant differences were determined using the Wilcoxon Rank Sum Test (wilcox.test in R3.6.1) using within-individual dissimilarity as the reference for comparisons.

### Genus differential abundance testing between disease groups

We used DESeq2 ^29^ to assess differential genus abundance between disease groups. This was performed in a pairwise manner between each disease group, in each tissue separately. Genera were deemed significantly differentially abundant at an adjusted p-value < 0.05.

### Host-microbiome correlation analysis

The method we employed for assessing host-microbiome correlations is outlined in Extended data Figure S3 ^38^. In total, we had matched RNA-seq and 16S rRNA amplicon sequencing data from 24 Ileal samples, 25 caecal samples and 24 rectal samples.

To remove the effect of disease on both host transcription and genus abundance, and thus the potential for spurious correlations, we first removed effects of disease status using Combat-seq ^41^. Processed count matrices were taken forward for downstream analysis. Host gene expression counts were normalised using the log2(counts per million) and these values were z-score scaled (across samples) to eliminate effects of expression level when defining co-expressed genes. Co-expression modules for each tissue location were defined using WGCNA ^42^ with default parameters. Each module in each tissue was assessed for enrichment of GO biological pathways using GOSeq ^43^. If significant enrichments were identified (padj < 0.05) then the module was annotated with the top pathway i.e. smallest adjusted p-value. Centred log-ratio transform using ALDEx2 ^44^ was used on genus counts and these were used for correlation analysis. Pearson product-moment correlations were used to correlate all module eigengenes (i.e. the first principal component of standardised expression levels for each module) and non-zero genus abundances using the *corr.test* function from psych ^45^. Correlations were retained if they reached an adjusted p-value < 0.05. As module eigengenes provide a representative expression summary for each module, we used significant correlations to conclude broad functional associations. Resulting networks were visualised using igraph v1.2.6 ^46^.

Analysis code can be found as extended data ^47^.

## Results

### Tissue-defining transcriptional signatures across intestinal tissue sites

The number of participants and sample characteristics are provided in Table 1.

There were no significant differences between groups for demographic variables (age and sex, Extended data Figure S1A ^38^). Significant differences were found for medication use (Extended data Figure S1B ^38^). These included significant differences in the proportion of individuals receiving ursodeoxycholic acid (URSO) between groups (PSC/UC (100%), UC (0%), healthy (0%), p = 6.4×10 ^-7^) and aminosalicylates (PSC/UC (100%), UC (80%), healthy (0%), p = 1.30×10 ^-6^). The majority of patients with either PSC/UC or UC did not have active colitis (Extended data Figure S1C ^38^) and there were no significant differences between patients with PSC/UC and patients with UC for neither the Nancy index (p = 0.26) nor the UCEIS (p = 1). While higher in a subset of patients with PSC/UC, liver function measures (Bilirubin, ALT and ALP) were not significantly different between groups (Extended data Figure S1D ^38^).

**Figure 1.**
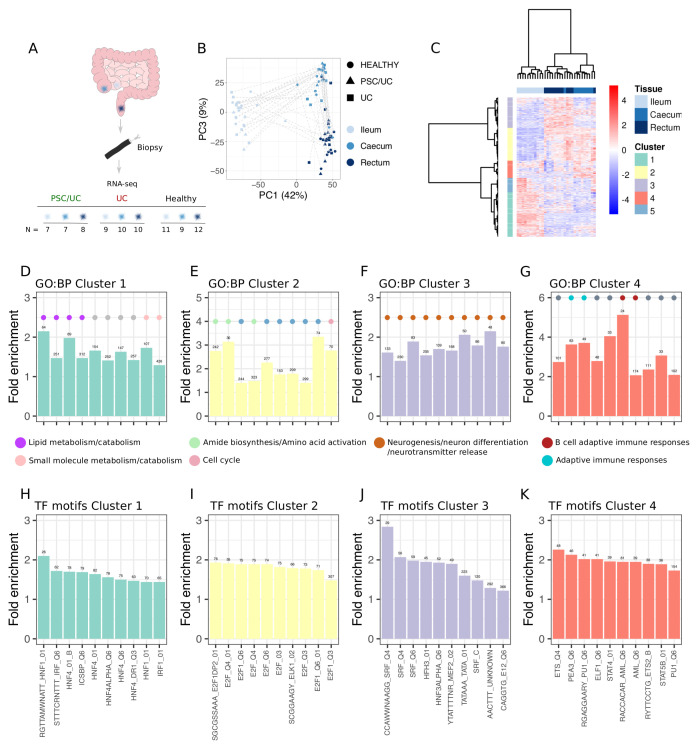
Tissue-defining transcriptional signatures. ( **A**) Experimental design and number of samples obtained for ribonucleic acid (RNA)-seq. ( **B**) Principal components analysis (PCA) showing the relationship between all samples (PCA performed on log2(counts per million) with the *prcomp* function in R3.6.1). ( **C**) Heatmap (Manhattan distance, Ward.D2 clustering) showing genes called as significantly differentially abundant across tissues in healthy individuals. Clusters defined using the *dynamicTreeCut* method are displayed as row annotations. ( **D**– **G**) Top 10 gene ontology biological pathways (GO:BP) that are significantly enriched (false discovery rate (FDR) < 0.05) amongst genes in each tissue-defining cluster. GO categories are coloured (filled circles) based on manual annotation of GO:BP. ( **H**– **K**) Top 10 transcription motif gene sets (MsigDB, C3, regulatory targets) that are significantly enriched (FDR < 0.05) amongst genes in each tissue-defining cluster.

Given there is known functional variation between intestinal locations, first we aimed to assess how global gene expression profiles vary by both tissue location and disease status. We generated paired RNA-seq data sets from biopsies taken from the ileum, caecum and rectum of patients with PSC/UC (n = 8), UC (n = 10) and healthy individuals (n = 12) ( Figure 1A). Principal components analysis (PCA) revealed tissue location as a major source of gene expression variation. Ileal samples separated from both caecal and rectal samples along principal component (PC) 1 (39%), with caecal and rectal samples displaying similar yet distinct transcriptional profiles (PC3, 9%) ( Figure 1B). To examine in more detail genes and gene sets that define different tissue locations, we performed differential expression analysis and identified 13,071 genes (60% of all genes analysed) as differentially expressed (Extended data Table S1 ^38^) between tissues in healthy individuals. These genes could be further partitioned into five clusters that reflected genes that were highest in the ileum (cluster 1), high in the caecum and rectum relative to the ileum (cluster 2), highest in the rectum (cluster 3), a mixed cluster that contained genes that were either high in the caecum or high in the ileum (cluster 4), and genes that were high in a subset of healthy ileal samples (cluster 5) ( Figure 1C and Extended data Table S2 ^38^). Ileum-defining genes (cluster 1, 3218 genes) were enriched for gene ontology biological pathways (GO:BP) associated with lipid and drug metabolism ( Figure 1D). Caecum/rectum-defining genes (cluster 2, 3082 genes) were enriched for GO:BP involved in cell cycle and amino acid activation ( Figure 1E). Rectum-defining genes (cluster3, 2853 genes) were significantly enriched for GO:BP involved in neurogenesis and neurotransmitter release ( Figure 1F). Cluster 4 genes (2271 genes) were enriched for GO:BP linked with adaptive immune responses ( Figure 1G). Finally cluster 5 (1647 genes) were not enriched for any GO:BP. Enrichment results are available as Extended data tables (Extended data Tables S3–6 ^38^).

Having mapped tissue-defining pathways, we reasoned that these functional differences would at least in part be driven by tissue-dependent transcription factor profiles. We explored this by assessing enrichment of transcription factor binding motifs amongst genes in each cluster. Each cluster was associated with specific transcription factors (Extended data Tables S7–10). Cluster 1 (ileum-defining) was associated with genes that are predicted to be regulated by hepatic nuclear factors (HNF) ( Figure 1H), cluster 2 (caecum/rectum-defining) with E2F transcription factors involved in the cell cycle ( Figure 1I), cluster 3 (rectum-defining) genes with serum response factor (SRF) transcription factors ( Figure 1J) and cluster 4 (mixed ileum and caecum) genes with erythroblast transformation specific (ETS) family transcription factors including PU.1 ( Figure 1K).

**Figure 2.**
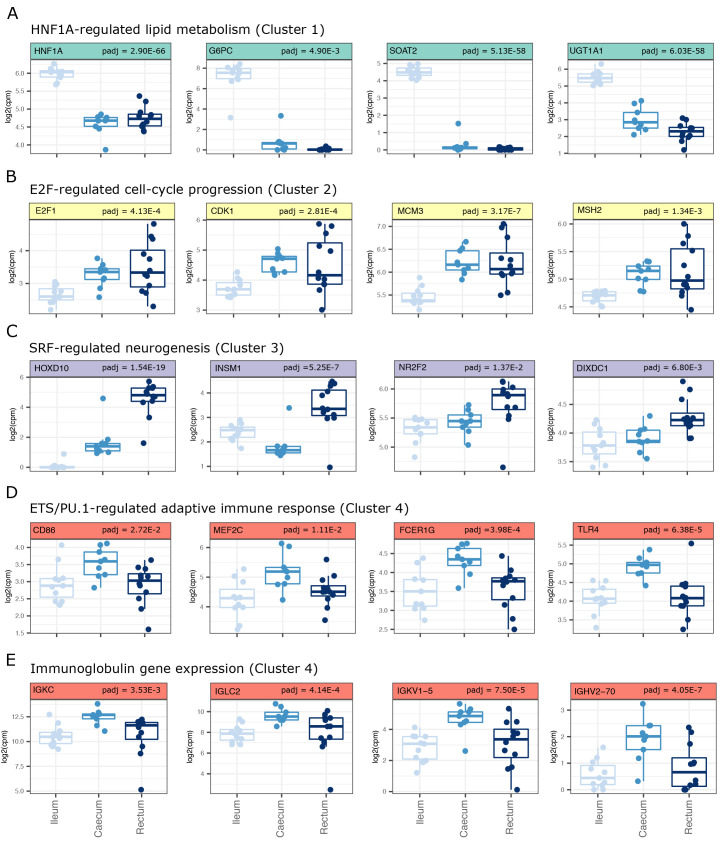
Tissue-defining gene expression signatures support distinct transcription factor profiles. ( **A**) Genes in cluster 1 are enriched for genes with HNF transcription factor motifs in their promoters. Genes plotted represent genes that were found in gene ontology biological pathways (GO:BP) annotated as being involved in lipid metabolism and also predicted to be regulated by HNF transcription factors (MSigDB, C3 regulatory targets gene sets). ( **B**) Genes in cluster 2 are enriched for genes with E2F transcription factor motifs in their promoters. Genes plotted represent genes that were found in GO:BP annotated as being involved in cell cycle progression and also predicted to be regulated by E2F transcription factors (MSigDB, C3 regulatory targets gene sets). ( **C**) Genes in cluster 3 are enriched for genes with SRF transcription factor motifs in their promoters. Genes plotted represent genes that were found in GO:BP annotated as being involved in neurogenesis and also predicted to be regulated by SRF transcription factors (MSigDB, C3 regulatory targets gene sets). ( **D**) Genes in cluster 4 are enriched for genes with ETS/PU.1 transcription factor motifs in their promoters. Genes plotted represent genes that were found in GO:BP annotated as being involved in adaptive immune responses and also predicted to be regulated by PU.1 transcription factors (MSigDB, C3 regulatory targets gene sets). ( **E**) Genes in cluster 4 that also contribute to a signal of adaptive immune responses are multiple immunoglobulin genes. Adjusted p-values (padj) values are based on the DESeq2 likelihood ratio test (LRT).

Consistent with the transcription factor binding predictions, we observed significant increased expression of hepatic nuclear factor 1A ( *HNF1A*) in the ileum (cluster 1), along with genes predicted to be regulated by HNF and involved in lipid metabolism ( *G6PC, SOAT2* and *UGT1A1*, Figure 2A). We also observed significantly increased expression of the E2F transcription factor, *E2F1*, that regulates key cell-cycle genes *CDK1, MCM3* and *MSH2* in caecal/rectal samples (cluster 2, Figure 2B). While SRF itself was not significantly up-regulated in rectal samples, SRF-regulated genes involved in neurogenesis including *HOXD10, INSM1, NRF2* and *DIXDC1* were associated with the rectal transcriptome (cluster 3, Figure 2C). The ETS family member PU.1 is known to regulate myeloid cell activation ^48^. We found predicted targets of ETS/PU.1 to have increased expression in caecal samples. These up-regulated genes included classical markers of myeloid cell activation ( *CD86, MEF2C, FCER1G* and *TLR4,* Figure 2D). These data suggested an increased antigen-driven cellular activation of myeloid cells in healthy caecum relative to ileum and rectum. Indeed, in addition to a proposed myeloid cell activation, we observed an increased expression of immunoglobulin genes ( Figure 2E) in caecal samples relative to ileum and rectum, supporting an increased proportion of activated B cells in this tissue.

Together, these data support tissue-dependent functions that reflect HNF-regulated lipid metabolism in the ileum, SRF-dependent neuronal function in the rectum and antigen-dependent lymphocyte activation in the caecum.

### Tissue-dependent changes in gene expression in PSC/UC and UC

Having established tissue-defining gene expression profiles across healthy intestinal sites, we next aimed to explore gene expression alterations in disease and how they map onto tissue signatures.

**Figure 3.**
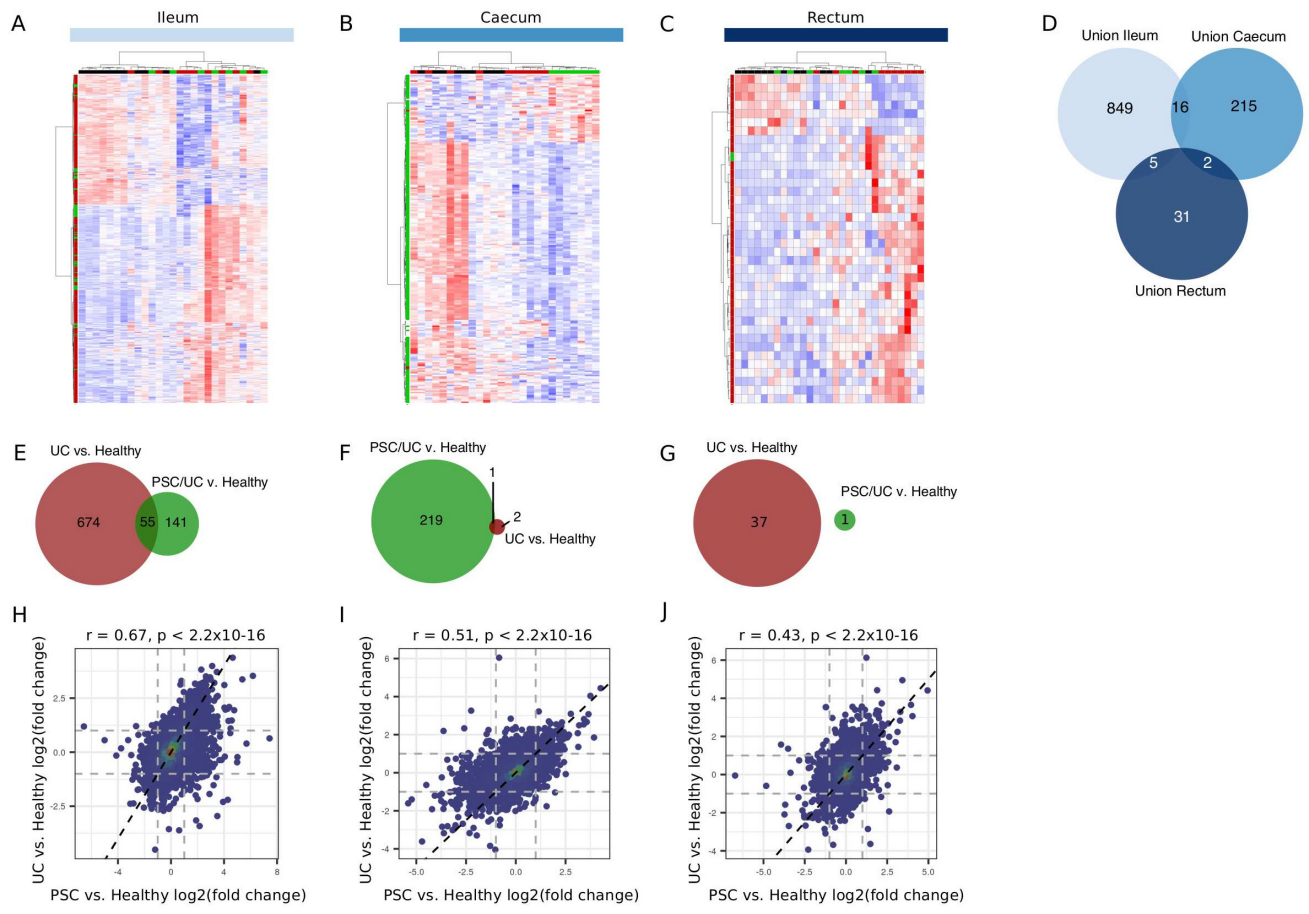
Differentially expressed genes in the ileum, caecum and rectum between disease and healthy. Heatmaps (Manhattan distance with Ward.D2 clustering) of log2(CPM) values for the union of differentially expressed genes (DESeq2 adjusted p-value < 0.05) in disease (either PSC/UC or UC) vs. healthy in ileum ( **A**), caecum ( **B**) and rectum ( **C**) are shown. ( **D**) Overlap between tissue sites for the union (as described) of disease-associated genes. Venn diagrams for the overlap of genes in PSC/UC vs healthy and UC vs. healthy in the ileum ( **E**), caecum ( **F**) and rectum ( **G**) are displayed. The overall correlation of effect sizes (log2(fold change)) between PSC/UC vs. healthy and UC vs. healthy in the ileum ( **H**), caecum ( **I**) and rectum ( **J**) are shown. Correlations and significance calculated using the Pearson correlation coefficient implemented in the *cor.test* function in R3.6.1. PSC/UC=primary sclerosing cholangitis-associated ulcerative colitis.

We determined gene expression differences between healthy individuals and disease (PSC/UC and UC) at each tissue location (Extended data Tables S11–16). A summary of the number of changes in each comparison is shown in Table 2 (false discovery rate (FDR) < 0.05). The total number of differentially expressed genes varied between each comparison ( Figure 3A–C), with differences being largely tissue dependent ( Figure 3D). While there was little overlap in genes differentially regulated in each disease relative to healthy individuals ( Figure 3E–G), effect sizes (i.e. log2(fold change)) were well correlated, particularly for effects observed in the ileum ( Figure 3H) and to a lesser extent in the caecum ( Figure 3I). In addition, and while correlated, effect sizes in the rectum were higher in UC when compared with healthy than PSC/UC compared to healthy ( Figure 3J). This is consistent with clinical features of these diseases, with rectal involvement predominantly being a feature of UC as opposed to PSC/UC.

**Table 2.** Number of genes called as significantly differentially expressed in each comparison (false discovery rate (FDR) < 0.05).

	Ileum (up)	Ileum (down)	Caecum (up)	Caecum (down)	Rectum (up)	Rectum (down)
**PSC/UC vs. healthy**	151	45	46	174	6	0
**UC vs. healthy**	427	302	1	2	30	7

**Figure 4.**
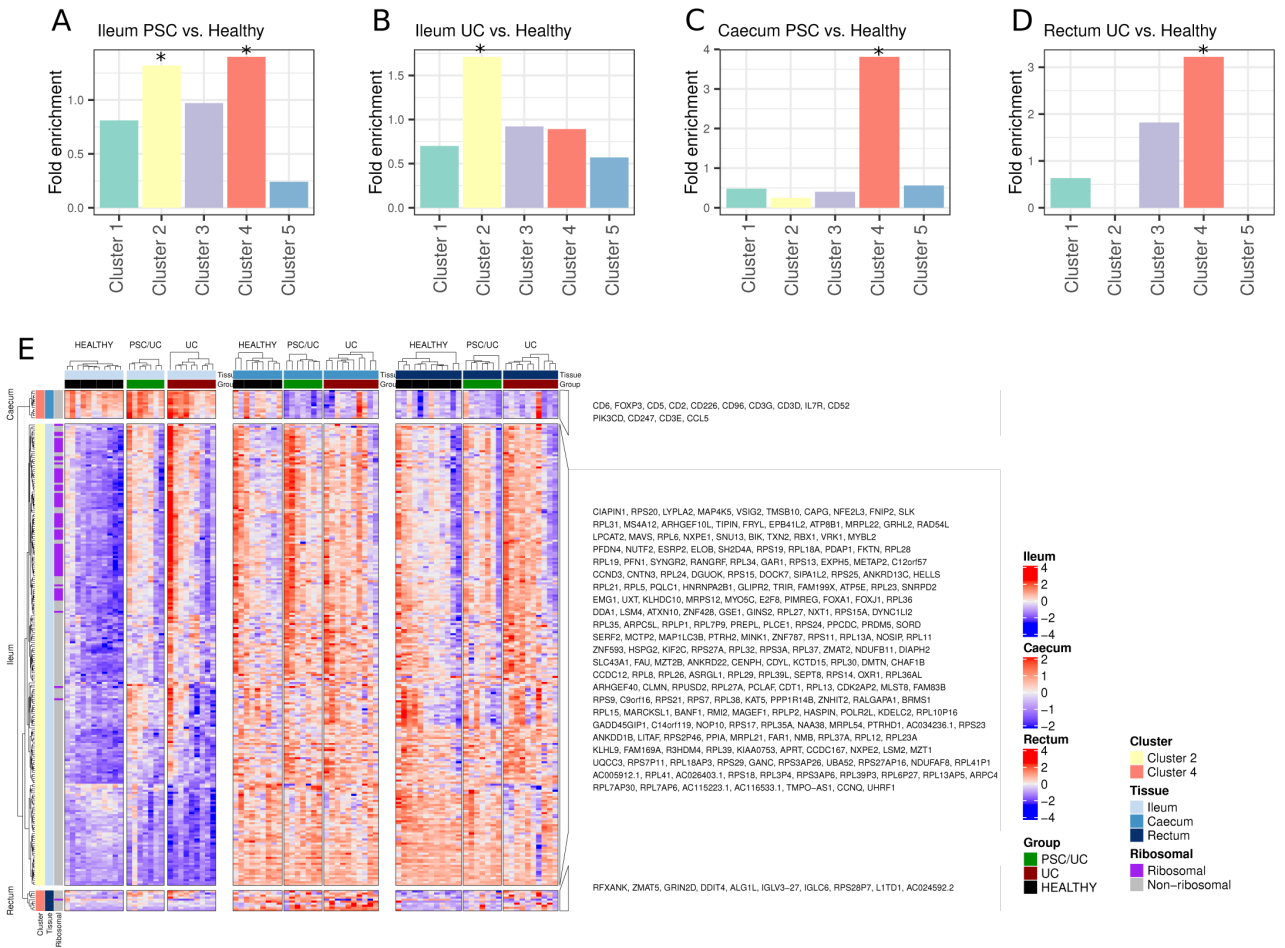
Enrichment of tissue-defining clusters amongst disease-associated genes. ( **A**– **B**) Genes identified as significantly differentially expressed between PSC/UC vs. healthy or UC vs. healthy in the ileum were tested for enrichment for each previously described tissue-defining cluster. Asterisks denote significant enrichment of a given cluster at a Benjamini-Hochberg adjusted p-value < 0.05 (hypergeometric test). ( **C**) Genes identified as significantly differentially expressed between PSC/UC vs. healthy in the caecum were tested for enrichment for each previously described tissue-defining cluster. No significant enrichment was found for genes differentially expressed in UC vs. healthy caecum and as such the plot is not shown. ( **D**) Genes identified as significantly differentially expressed between UC vs. healthy in the rectum were tested for enrichment for each previously described tissue-defining cluster. No significant enrichment was found for genes differentially expressed in PSC/UC vs. healthy rectum and as such the plot is not shown. ( **E**) Heatmap visualisation of significantly differentially expressed genes between PSC/UC vs. healthy or UC vs. healthy that belong to significantly enriched clusters at each tissue site. Clusters are provided as left annotations and the tissue site where differential expression was observed is also annotated. Heatmaps were produced using the ComplexHeatmap package ^54^ in R3.6.1. As numerous ribosomal proteins were observed to be differentially expressed in the ileum, these are also annotated. PSC/UC=primary sclerosing cholangitis-associated ulcerative colitis.

Given a high degree of tissue dependency on disease-associated gene expression, we wanted to explore whether this was due to inherent differences in tissue gene expression. To explore this, we utilised our previously identified tissue-defining gene clusters to define overrepresented tissue-dependent processes in disease-associated gene lists. In the ileum, disease-associated transcriptional changes were not associated with ileum-defining transcriptional signatures in general. Indeed, changes in both PSC/UC and UC were enriched for genes that typically define colonic tissue sites (Clusters 2 and 4, Figure 4A and B). For example, the expression of ribosomal proteins that define a caecum/rectum profile (cluster 2) were increased in expression in both PSC/UC and UC when compared to healthy controls ( Figure 4E). These changes may be partially explained by an increase in proliferating lymphocytes in disease, as we also observed an increase in expression of the lymphocyte-specific helicase, *HELLS* ( Figure 4E), particularly in PSC/UC. In the caecum, PSC/UC-associated changes were enriched for genes belonging to cluster 4 ( Figure 4C). These changes are associated with immune signatures that appear to define T cell alterations in PSC/UC, and to a lesser extent UC. Such changes are exemplified by reduced expression of key T cell markers including *CD2*, *CD3D, CD3E* and *CD3G* ( Figure 4E). These general changes may reflect alterations in specific T cell populations as both *IL7R* and *FOXP3* ( Figure 4E) were reduced in PSC/UC. To further explore whether there was a reduction in specific immune cell populations we performed cell deconvolution analysis using CIBERSORTx ^32^. Prediction of cellular fractions from 25 human gut cell types revealed no statistically significant differences in cellular composition between disease groups in any of the three tissue types. Further work using single cell measurements with either flow cytometry or single cell RNA-seq would be required to accurately deconvolve this signal.

In UC, changes in the rectum relative to healthy individuals were enriched for genes in cluster 4 ( Figure 4D) and include *ALG1L*, *RFXANK*, *L1TD1* and *ZMAT5* ( Figure 4N–Q). These changes represent a relatively broad altered functional profile that includes increased expression of genes involved in glycosylation ( *ALG1L1*), self-renewal ( *L1TD1*) ^49^ and MHC class II processing ( *RFXANK*) ^50^.

### Gamma-glutamyl transferase 1 ( *GGT1*>) is specifically reduced in PSC/UC

**Figure 5.**
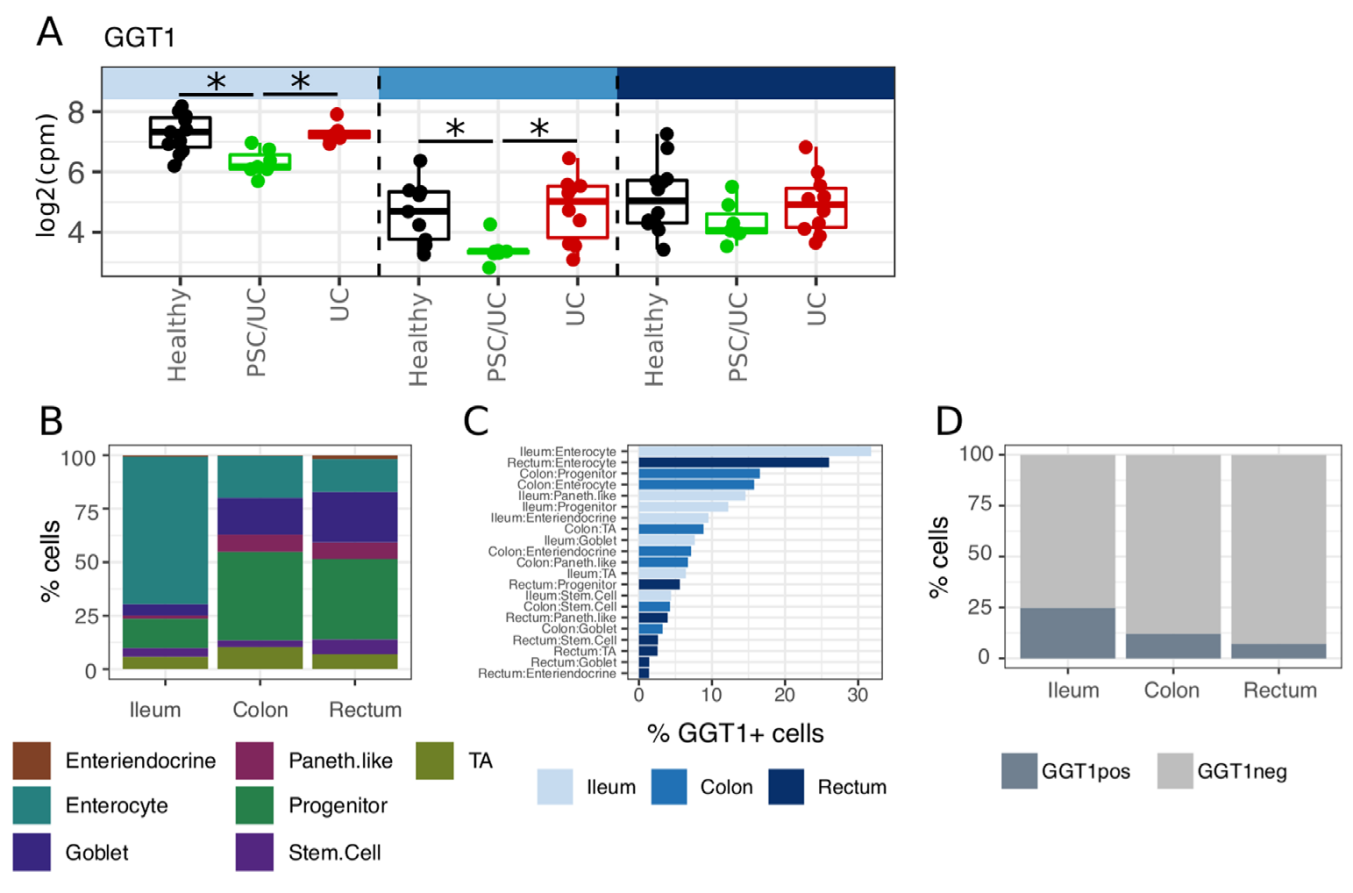
Gamma-glutamyl transferase 1 ( *GGT1*) is specifically downregulated in PSC/UC ileum and caecum. ( **A**) Differential expression of *GGT1* across tissue sites. Asterisks denote DESeq adjusted p-value < 0.05. ( **B**) Single-cell ribonucleic acid (RNA)-seq data from a publicly available data set (GEO accession GSE125970) supports an enrichment of enterocytes in the ileum relative to colon and rectum. ( **C**) *GGT1+* cells (defined as cells with > 0 mapping to *GGT1*) are particularly enriched in ileum enterocytes that contributes to the increased frequency of *GGT1+* cells in the ileum relative to the colon and rectum ( **D**). PSC/UC=primary sclerosing cholangitis-associated ulcerative colitis.

We directly compared PSC/UC to UC to identify genes that were specifically altered in PSC/UC. In total, three, sixteen, and nine genes were differentially expressed in ileum, caecum and rectum, respectively (Extended data Tables S17–19). Of these, *GGT1* was significantly reduced in both the ileum and caecum in patients with PSC/UC relative to both healthy individuals and patients with UC ( Figure 5A). In contrast to the ileum and caecum, we did not observe a significant reduction in *GGT1* expression in the colon of patients with PSC/UC in an independent, publicly available data set ^8^ suggesting that this alteration is specific to the small intestine and proximal colon.

Increased serum gamma-glutamyl transferase (GGT) is a diagnostic marker of alcoholic liver disease ^51^ and cholestasis in children ^52^. Functionally, it participates in the glutathione salvage pathway, enabling glutathione-dependent reduction of reactive oxygen species in the cell. In the kidneys, reduced *GGT1* expression is linked to mitochondrial dysfunction and metabolic re-wiring in a relatively rare renal cancer - Chromophobe renal cell carcinoma (ChRCC) ^53^. We therefore reasoned that this enzyme may be an important modulator of colon cancer risk in PSC/UC and as such would be expressed in intestinal epithelium. To examine this, we leveraged publicly available single cell RNA-seq data generated from the epithelial compartment of human ileum, colon and rectum ^10^. As described in the original publication, the ileal epithelium differed substantially from the colonic and rectal epithelium in cellular composition, particularly in terms of an increased proportion of enterocytes ( Figure 5B). Cells expressing *GGT1* ( *GGT1+* = >= read) were enriched in enterocytes ( Figure 5C), a feature that contributed to an observed increase in proportion of *GGT1+* cells in the ileum when compared to the colon and rectum ( Figure 5D). These data support expression of GGT1 in the enterocyte compartment of the ileal and colonic epithelium. A defining feature of PSC/UC compared with UC is liver disease and cholestasis. Given the strong functional links between the liver and the intestine, particularly with respect to bile acid secretion ^55^, our results suggest that further work should aim to identify links between altered liver function and downstream effects on intestinal *GGT1* expression in patients with PSC/UC.

### Mucosa-associated bacterial associations with disease

**Figure 6.**
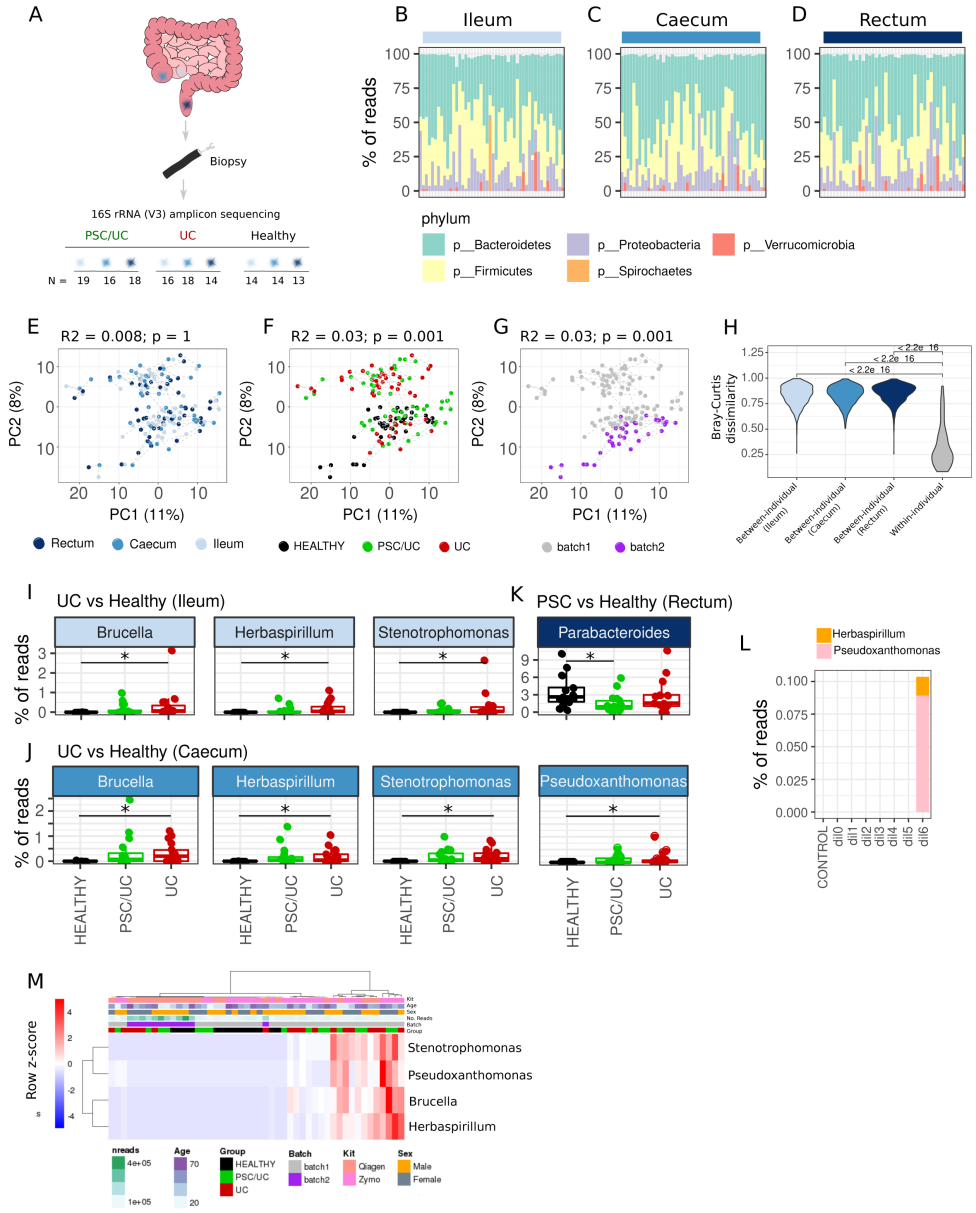
Mucosa-associated bacterial associations in PSC/UC and UC. ( **A**) Description of the number of samples collected for 16S ribosomal ribonucleic acid (rRNA) amplicon sequencing. High-level phylum-level classification and abundances are provided for the ileum ( **B**), caecum ( **C**) and rectum ( **D**). Principal components analysis (PCA) plots of log10(relative abundance + 1E-6) at the genus level showing clustering by tissue ( **E**), disease group ( **F**) and sequencing batch ( **G**) are shown. R ^2^ and p-values were computed using the *adonis* test in the *vegan* package in R3.6.1. For the assessment of both tissue and disease associations, sequencing batch was included as a covariate. ( **H**) Violin plot displaying inter- and within-individual dissimilarity measured by Bray-Curtis dissimilarity on relative abundance estimates. Bray-Curtis dissimilarity was computed using the *Phyloseq* package in R3.6.1. ( **I**) Differentially abundant (DESeq2 adjusted p-value < 0.05) genera in UC vs. healthy ileum. ( **J**) Differentially abundant (DESeq2 adjusted p-value < 0.05) genera in UC vs. healthy caecum. ( **K**) Differentially abundant (DESeq2 adjusted p-value < 0.05) genera in PSC/UC vs. healthy rectum. Asterisks denote DESeq2 adjusted p-value < 0.05. ( **L**) Detection of likely contaminants in control samples. CONTROL = Endoscope control, dil = dilution from dil0 (caecal contents 1:1) – dil6 (caecal contents 1:1,000,000). ( **M**) Heatmap showing the distribution of potential contaminants amongst caecal samples. Row-scaled relative abundances are shown and metadata are labelled. The heatmap was produced using pheatmap with rows and columns clustered using Manhattan distance and Ward.D clustering. PSC/UC=primary sclerosing cholangitis-associated ulcerative colitis.

The intestinal microbiota performs essential homeostatic roles and is an important mediator of the liver-gut axis that includes the deconjugation of liver-derived bilirubin and bile acids. Given functional differences and microbiome composition differences between intestinal sites, we aimed to assess bacterial associations in PSC/UC of the mucosa-associated microbiota across the ileum, caecum and rectum. We performed 16s rRNA amplicon sequencing ( Figure 6A) of biopsy samples from the same three tissue sites as described (ileum, caecum and rectum) in healthy individuals, patients with PSC/UC and patients with UC.

Following a similar approach as previously described for assessing potential reagent contamination ^39^, using serially diluted (1:1 – 1:1000,000) mouse caecal content samples from mice with a defined 12-member community ^37^ we observed little evidence for reagent contamination. Indeed, between 96.3% and 100% of sequenced reads were assigned to genera within this community (Extended data Figure S2 ^38^). All expected genera were recovered from this community, supporting a reasonable extraction and sequencing approach in this study. Nevertheless, using this approach, as well as sequencing data from an endoscope control (negative control), we identified 135 ASVs that we considered as potential contaminants (Extended data Table S20 ^38^). While these sequences were retained for downstream analysis, we used them as a reference when reviewing significant disease associations.

Samples were removed from the analysis if they had fewer than 10000 reads. The mean number of reads remaining after filtering for host-associated reads and processing with dada2 was 95617 (10542–543905). On average, we could assign 99% of sequence reads to five phyla, with all samples dominated by three major phyla typical of intestinal microbiomes (Firmicutes, Bacteroidetes and Proteobacteria, Figure 6B–D). Using amplicon sequence variant (ASV) abundances, we observed no significant association between disease status and Shannon diversity in the ileum (F(2,45) = 1.90, p = 0.16), caecum (F(2,44) = 1.88, p = 0.17, or rectum (F(2,41) = 0.16, p = 0.85)). Further downstream analyses were performed at the genus level i.e. ASV counts summed at the level of genus (total number of genera analysed was 128). In contrast to host transcriptomes, overall microbiome composition was not associated with tissue location ( Figure 6E). However, there was a significant clustering by disease status ( Figure 6F) and sequencing batch ( Figure 6G). Nevertheless, the variation in the bacterial component of the microbiome was driven by inter-individual differences. Indeed, samples from the same individual (across tissue locations) were more similar to each other than samples from the same tissue location (between individuals) ( Figure 6H). In differential abundance analysis of individual genera, we identified three genera as significantly differentially abundant between patients with UC and healthy controls in the ileum (adjusted p < 0.05, *Brucella, Herbaspirillum* and *Stentrophomonas*, Figure 6I) and four in the caecum ( *Brucella, Herbaspirillum, Stentrophomonas* and *Pseudoxanthomonas,* Figure 6J), three of which overlapped between tissue sites ( *Brucella, Herbaspirillum* and *Pseudoxanthomonas*). One genus was found to be differentially abundant in the rectum ( *Parabacteroides*, Figure 6K) between patients with PSC/UC and healthy controls (Extended data Tables S21–26). Given the relatively low prevalence and low abundance of *Brucella*, *Herbaspirillum*, *Stenotrophomonas* and *Pseudoxanthomonas* we examined whether there was evidence that these were contaminants. *Herbaspirillum*, *Stenotrophomonas* and *Pseudoxanthomonas* have been reported as reagent contamination artefacts in previous studies ^38^. Using our control data, we were able to identify *Herbaspirillum* and *Pseudoxanthomonas* as likely contaminants. Indeed, these genera were identified in the most dilute samples of our known microbial community controls ( Figure 6L). We did not observe *Brucella* nor *Stenotrophomonas* in our control data sets. However, the observation that their abundance covaried with likely contaminants, as well as them only appearing in one of the two sequencing batches ( Figure 6M), suggests that they are part of the same contaminant profile.

Together these data support high inter-individual variability in mucosa-associated bacterial composition and little evidence for tissue- or disease-associated bacterial genera. Nevertheless, there is a decreased abundance of *Parabacteroides* in the rectum of patients with PSC/UC. 

### Exploring host-bacteria associations across tissue sites

Next, we sought to understand the relationship between host gene expression and bacterial relative abundances at each tissue site. To assess this, we correlated bacteria relative abundances with host gene expression modules derived from weighted gene co-expression network analysis (WGCNA). We identified 22, 28 and 23 host modules in the ileum, caecum and rectum, respectively. Of these, 9 (40%), 20 (71%) and 16 (70%) could be annotated with at least one significantly enriched (padj < 0.05) GO:BP in the ileum, caecum and rectum, respectively.

**Figure 7.**
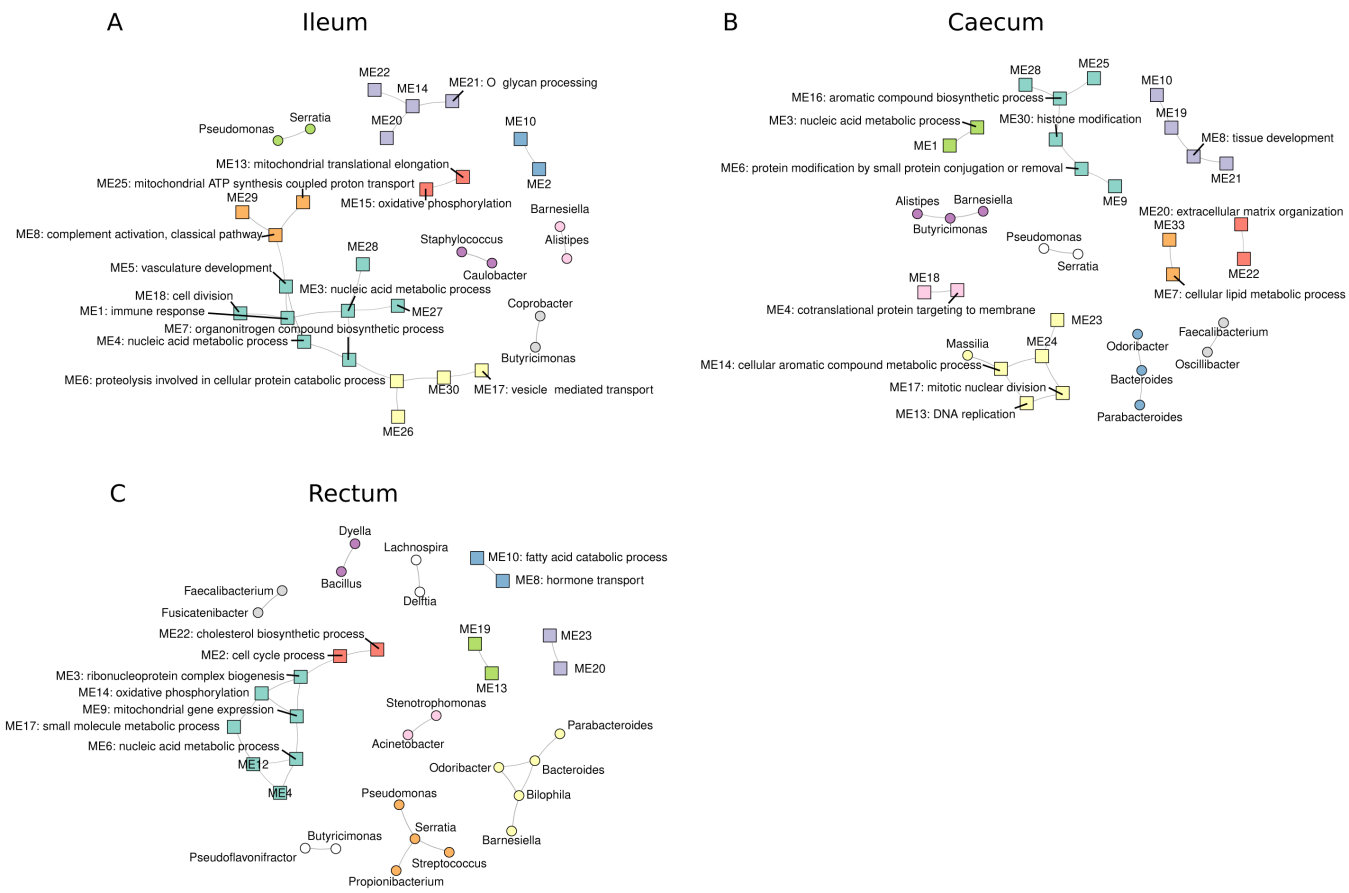
Host-bacteria associations in the intestine. Correlation networks in ileum ( **A**), the caecum ( **B**) and the rectum ( **C**) were built by correlating (Pearson product-moment correlation) non-zero, centred log ratio (CLR)-transformed genus counts with module eigengenes from the respective tissue gene expression data. Graphs were generated using the igraph package V1.2.6. Shapes of vertices are dependent in their origin i.e. square = host eigengene and circle = genus. Membership of each vertex to a cluster (coloured) was determined using the cluster_fast_greedy function in igraph. CPM=counts per million. CLR=centred log-ratio.

Correlation analysis between all module eigengenes and centred log-ratio (CLR)-transformed genus counts revealed an influence of high numbers of zero values in genus abundances in driving significant host-bacteria correlations. For example, we observed a statistically significant (Benjamini-Hochberg adjusted p < 0.05) correlation between *Corynebacterium* abundance and a host module eigengene, ME25 (Extended data Figure S4A). However, due to the large number of zero counts in these data (Extended data Figure S4B), it was evident that this significant correlation was being driven by CLR-transformed zero counts (Extended data Figure S4C) leading to the conclusion that this was a spurious correlation. To counter this effect, we re-ran the correlation analysis using only non-zero data. These analyses revealed little evidence for strong host-bacterial associations. Indeed, in all tissue sites, statistically significant (Benjamini-Hochberg adjusted p < 0.05) correlations were restricted to intra-host or intra-bacterial correlations ( Figure 7A–C). Our data suggest that host gene expression is not strongly associated with genus abundance.

## Discussion

The human intestine is a multi-functioning organ that performs tasks from nutrient absorption and metabolism to protection against invading pathogens. In this study we have expanded on existing knowledge regarding the distribution of functions across different sites in the human intestine. In healthy individuals we observed increased expression of genes involved in lipid and small molecule metabolism in the ileum relative to both the caecum and rectum. These results were expected given previous observations of increased expression of genes involved in lipid assimilation in small intestinal epithelial cells relative to the colon and rectum ^10^. Indeed, and while not discussed in detail here, we observed similar increased expression of *APOA1* and *APOM* in the ileum, as described previously ^10^. We identified *HNF* transcription factors as key markers of ileal transcriptomic signatures. In addition to enrichment of *HNF* motifs among genes more highly expressed in the ileum relative to the caecum and rectum, we identified increased expression of *HNF1A* in the ileum. *HNF1A* is a key transcription factor in multiple metabolic processes across multiple cell types, with mutations in *HNF1A* causing maturity-onset diabetes of the young (MODY) ^56^. Our data corroborate previous experiments in animal models suggesting that in addition to its role in glucose metabolism it has a prominent role in lipid metabolism ^57^.

Both the caecum and the rectum were characterised by increased expression of genes related to cell cycle progression. Indeed, we observed an increased expression of the transcription factor *E2F1* in the caecum and rectum relative to the ileum. E2F1 promotes cell cycle progression into S phase ^58^ through its regulation of replication-promoting target genes such as *CDK1* ^59, 60^, a gene that we also observed to have increased expression in both the caecum and rectum relative to the ileum. These data support an increased frequency of proliferative cells in the colon relative to the small intestine. While we cannot determine the specific cell types that are proliferating from our data, it is likely that increased frequencies of proliferative stem, transit-amplifying, and progenitor epithelial cells ^61^ in the colon relative to the ileum ^10^ contribute at least in part to our observations in bulk tissue samples.

We were able to further identify a cluster (cluster 4) of genes that represented transcription of immune processes – particularly adaptive immunity. While this cluster was somewhat mixed in terms of tissue distribution, we were able to identify transcriptional signatures of immune activation that were predominantly associated with increased transcription in the caecum relative to the ileum and rectum. These genes included *CD86*, *MEF2C, FCER1G* and *TLR4*. CD86 is expressed on the cell surface of antigen presenting cells (APCs) including dendritic cells, macrophages and B-cells, and participates in the activation of adaptive immunity through T-cell activation ^62^. The increased expression of this marker in the caecum supports antigen-induced activation at this tissue site ^63^. Further support for increased antigen-dependent activation of APCs in the caecum comes from observations of increased expression of *FCER1G* and *TLR4*, genes known to be involved in mediating innate control of adaptive immunity ^64^. The interactions of APCs with microbial antigens in the caecum could also explain increased expression of immunoglobulin genes in the caecum, which we propose represent an enrichment of activated B cells in the caecum relative to the ileum and rectum.

Together with the central nervous system (CNS), the enteric nervous system (ENS) is essential for digestion through controlling peristalsis and movement of fluids across the mucosa ^65^. We identified an enrichment of genes involved in neurogenesis that are more highly expressed in the rectum relative to both the ileum and caecum. Control of these genes was, at least in part, explained by an enrichment of serum response factor (SRF) motifs in the promoter regions of these genes. It is difficult to fully interpret these results as although SRF is a known neuroprotective factor in cortical neurons ^66^, our data do not support a general increase in neurons in the rectum relative to the ileum and caecum. For example, we do not see elevated expression of the neuronal marker *ELAVL4* in the rectum. In contrast, we did observe an increased expression of the enteric glial cell (EGC) marker, *GFAP* ^67^, in the rectum relative to both the ileum and caecum. Along with increased expression of the neuroendocrine monoamine transporter gene ( *SLC18A1*) and multiple genes involved in synapse formation (e.g., *SNAP25*, Synaptogagmins, Extended data Table 1), we would hypothesise that there is increased ENS-EGC crosstalk in the rectum relative to the ileum and caecum. Further work is required to determine if this is the case and what the functional consequences of such differences are.

One of the major aims of this study was to identify transcriptional profiles that vary across disease states and whether these are tissue-dependent. Differential expression analysis revealed multiple transcriptional differences between healthy individuals and individuals with either PSC/UC or UC across all three tissue sites. The majority of changes were identified in the ileum, with a substantial overlap between PSC/UC and UC. This is a noteworthy observation as patients with UC rarely present with ileitis although it can be detected endoscopically in some cases ^68^. In the caecum, patterns of gene expression were consistent with clinical differences between PSC/UC and UC. Although effect sizes between PSC/UC vs. healthy and UC vs. healthy were fairly well correlated, we observed an expected increased effect size in PSC/UC caecum relative to UC caecum. This is consistent with more proximal involvement in PSC/UC relative to UC. In contrast, in the rectum, increased effect sizes and thus differential features were observed in UC relative to PSC/UC. These data are consistent with increased rectal involvement in UC relative to PSC/UC. It was noteworthy that differences in transcription between either PSC/UC or UC vs. healthy were not conserved across tissue sites. Initially we hypothesised that this reflected underlying differences between tissues that we had described previously. However, we found that changes in the ileum were not significantly enriched in genes that define the ileum relative to the caecum and rectum. Indeed, genes that are more highly expressed in either PSC/UC or UC relative to healthy controls were enriched for genes that are in cluster 2 of tissue-defining features – proliferation related genes that are more highly expressed in the healthy caecum and rectum relative to the ileum. These changes consisted of multiple genes involved in protein synthesis, including multiple ribosomal proteins. We believe that these changes represent an increased in the frequency of proliferative immune cells in the ileum in patients with PSC/UC and UC. The higher expression of *HELLS*, a lymphocyte specific helicase, supports an increase in proliferating lymphocytes in the ileum, particularly in patients with PSC/UC. The increases in ribosomal proteins that we see also support cell growth/proliferation of immune cells. This is evident from cell-type specific expression patterns of a number of ribosomal proteins including *RPL36*, where expression is highest in T and B lymphocytes ^69^. Notably, we observed an increased expression of *RPL5*, a gene whose protein product is associated with P53-dependent and independent tumour suppressor function ^70, 71^. While the role of *RPL5* in reducing proliferation is incongruous with our data, recent evidence supports a role for P53 in regulating DNA polymerase restart during DNA replication ^72^, which may or may not involve RPL5.

We identified a clear T cell expression phenotype in the caecum of patients with PSC/UC relative to healthy controls. These changes may represent a reduced frequency of T cells as classical T cell markers such as *CD2, CD3E, CD3F* and *CD3G* were reduced in expression. Reduction in additional markers of T cell subsets including *IL7R* and *FOXP3* also point to a reduction in specific functional subsets, particularly in PSC/UC. However, we did not observe a significant association with any predicted cell fraction using cell deconvolution analysis. This may reflect complexity in such analyses, that cannot successfully decompose individual cellular expression from expression governed by cellular composition. Further work would be required to capture single cell measurements of gene or protein expression to confirm our data.

Rectal involvement is more frequent in UC compared to PSC/UC. This clinical feature was borne out in the results of our differential expression analysis whereby a greater number of differential features with larger effect sizes were identified in the UC vs. healthy comparison relative to the PSC/UC vs. healthy comparison. Differentially expressed genes in the rectum of patients with UC represent a diverse set of functions. These include glycosylation, self-renewal and MHC class II processing. It is not clear how these genes might contribute to pathology in UC although it is noteworthy that increased expression of *ALG1L1* in the rectum has been reported previously ^73^. Interestingly *L1TD1* is associated with self-renewal and the potential to promote cancer. Nevertheless, a recent report suggested that increased *L1TD1* expression is associated with increased survival in colorectal cancer ^49^. Further work is required to determine any functional impact of increased *L1TD1* in the rectum of patients with UC. 

A prominent clinical difference between PSC/UC and UC is a significant increased risk of colorectal cancer in patients with PSC/UC relative to UC ^3^. Pathways underlying this difference remain unclear, although potential mechanisms could involve altered tumour-promoting immune phenotypes, DNA damage pathways and/or changes in the abundance of carcinogenic microbes. We observed a significant reduced expression of *GGT1* in the ileum and caecum of patients with PSC/UC relative to both healthy individuals and patients with UC. This finding provides a compelling hypothesis linking liver function to intestinal redox homeostasis and colon cancer development. Indeed, elevated serum GGT is often used as a marker of cholestasis and liver dysfunction. Its role in liver function is also evident from recent observations of single nucleotide variation at the *GGT1* locus being associated with PSC ^74^. Our observations extend altered GGT function to the intestine. GGT1 is an important enzyme in the glutathione salvage pathway, degrading extracellular glutathione into its amino acid constituents for cellular uptake. Once inside the cell, glutathione is reformed and participates in the reduction of reactive oxygen species ^75^. Using publicly available data, we have shown that *GGT1* positive cells are present in the epithelial compartment of the small and large intestine. The predominance of *GGT1* positive cells in enterocytes appears to lead to an increase in *GGT1* positive cells in the ileum relative to the colon and rectum. This is noteworthy, as it suggests developmental differences between tissue sites that we believe may relate to the availability of liver-derived factors. We speculate that reduced bile flow has an impact on *GGT1* expression in the intestine. In this regard it is of interest that increased serum bilirubin has been associated with an increased risk of cholangiocarcinoma in patients with PSC ^76^. Whether this is also true for colon cancer development remains to be explored. Together these data support a new hypothesis of colon cancer development in PSC/UC that involves a liver dysfunction-induced deficit in the ability of intestinal epithelial cells to combat oxidative stress in the context of inflammation. We speculate that this may be a key driver of colon cancer development through increased DNA damage or metabolic dysfunction as has been shown for ChRCC ^53^.

In addition to host factors in PSC/UC, the microbiome has become increasingly recognised as an important contributory factor. Indeed, members of the microbiome are important mediators of the gut-liver axis through their ability to metabolise bile acids that reach the intestine from the liver. Multiple studies have provided links between the bacterial component of the microbiome from both stool ^12, 13, 77^ and mucosa ^17, 18^ with PSC/UC. Furthermore, mouse studies have provided a potential causal link between PSC-associated microbial consortia and Th17-driven immunopathology in both the gut and the liver ^20^. We did not observe any striking effects of the bacterial component of the microbiome across three tissue locations in patients with PSC/UC. After accounting for the presence of contaminants in our data set, we observed a single genus, *Parabacteroides*, as being significantly reduced in the rectum of patients with PSC/UC. This observation is consistent with a similarly reduced relative abundance of *Parabacteroides* in neonatal cholestasic disease ^78^. While not consistently observed in PSC, there has also been a report of increased *Parabacteroides* in the stool of patients with PSC ^77^. While contradictory in direction to the current study, differences in sampling method may be a relevant factor. Further work is required to fully disentangle genuine microbiome signals from these studies in the context of multiple influencing factors that may vary across study cohorts.

Finally, we show limited evidence for associations between genera abundances and expression of host genes. Previous studies have observed relationships between bacterial abundance and host gene expression ^79^ and we cannot discount that we simply did not have the power to detect robust associations in our relatively small cohort, especially given the sparseness of the data under consideration. An important observation from our data is the potential of CLR-transformed zero counts to drive host-microbiome associations. Zero counts are a known issue when using log-ratio transforms for microbiome data ^44, 80^ and while the ALDEx2 CLR transform deals well with zero counts we could not exclude that spurious correlations were introduced due to zero-count transformation. Using non-zero data for correlation analysis will likely have impacted our ability to identify robust host-microbiome associations and is a limitation of this study.

In conclusion, we have shown tissue-dependent gene expression differences across intestinal diseases and identified an association between reduced *GGT1* expression in the ileum and caecum of patients with PSC/UC that we hypothesise to link liver function with colorectal cancer risk in the context of inflammatory bowel disease.

## Data Availability

ArrayExpress: RNA seq data. Accession number E-MTAB-9658; https://identifiers.org/arrayexpress:E-MTAB-9658. 16S rRNA sequencing data have been deposited in the EMBL-EBI: 16S rRNA sequencing data. Accession number PRJEB40737; https://identifiers.org/ena.embl:PRJEB40737. Analysis code available from: https://github.com/nickilott/PSC_UC_Host_Microbiome Archived analysis code at time of publication: https://doi.org/10.5281/zenodo.4808793 ^47^. License: MIT Figshare: Tissue-dependent transcriptional and bacterial associations in primary sclerosing cholangitis-associated inflammatory bowel disease. https://doi.org/10.6084/m9.figshare.c.5438892.v2 ^38^. This project contains the following extended data: Figure S1 (Association of demographic, medication use, inflammation parameters and liver function with disease status.) Figure S2 (Relative abundance of a known caecal microbiome community across serially diluted caecal samples.) Figure S3 (Schematic overview of generating host-microbiome correlation networks. Figure S4 (Correlation network generated before accounting for a role of zero counts in genus CLR-transformed counts. Zero counts are shown to impact observed associations between host gene expression and genus CLR.) Table S1 (DESeq2 differential expression results in an LRT analysis between tissue sites in healthy individuals.) Table S2 (Cluster assignments of genes to tissue-defining modules using dynamicTreeCut.) Table S3 (GO:BP enrichment results for genes in cluster 1.) Table S4 (GO:BP enrichment results for genes in cluster 2.) Table S5 (GO:BP enrichment results for genes in cluster 3.) Table S6 (GO:BP enrichment results for genes in cluster 4.) Table S7 (Transcription factor enrichment results for genes in cluster 1.) Table S8 (Transcription factor enrichment results for genes in cluster 2.) Table S9 (Transcription factor enrichment results for genes in cluster 3.) Table S10 (Transcription factor enrichment results for genes in cluster 4.) Table S11 (DESeq2 differential expression results for Tissue: Ileum, contrast: PSC/UC vs. healthy.) Table S12 (DESeq2 differential expression results for Tissue: Ileum, contrast: UC vs. healthy.) Table S13 (DESeq2 differential expression results for Tissue: Caecum, contrast: PSC/UC vs. healthy) Table S14 (DESeq2 differential expression results for Tissue: Caecum, contrast: UC vs. healthy.) Table S15 (DESeq2 differential expression results for Tissue: Rectum, contrast: PSC/UC vs. Healthy) Table S16 (DESeq2 differential expression results for Tissue: Rectum, contrast: UC vs. healthy) Table S17 (DESeq2 differential expression results for Tissue: Ileum, contrast: PSC/UC vs. UC) Table S18 (DESeq2 differential expression results for Tissue: Caecum, contrast: PSC/UC vs. UC) Table S19 (DESeq2 differential expression results for Tissue: Rectum, contrast: PSC/UC vs. UC.) Table S20 (Putative contaminant ASVs.) Table S21 (DESeq2 genus differential abundance results for Tissue: Ileum, contrast: PSC/UC vs. healthy.) Table S22 (DESeq2 genus differential abundance results for Tissue: Ileum, contrast: UC vs. healthy.) Table S23 (DESeq2 genus differential abundance results for Tissue: Caecum, contrast: PSC/UC vs. healthy.) Table S24 (DESeq2 genus differential abundance results for Tissue: Caecum, contrast: UC vs. healthy.) Table S25 (DESeq2 differential expression results for Tissue: Rectum, contrast: PSC/UC vs. healthy.) Table S26 (DESeq2 differential expression results for Tissue: Rectum, contrast: UC vs. healthy.) counts.zip (Processed counts tables for RNA-seq and 16S amplicon sequencing data.) annotations.zip (Annotation files for reproducing data analysis.) metadata.zip (Metadata files for reproducing data analysis.) Figshare: ARRIVE checklist for ‘Tissue-dependent transcriptional and bacterial associations in primary sclerosing cholangitis-associated inflammatory bowel disease’. https://doi.org/10.6084/m9.figshare.c.5438892.v2 ^38^. Data are available under the terms of the Creative Commons Attribution 4.0 International license (CC-BY 4.0).
