# Supplementary material for: Tissue-dependent transcriptional and bacterial associations in primary sclerosing cholangitis-associated inflammatory bowel disease
Source: Wellcome Open Res. 2022 Nov 4;6:199. Originally published 2021 Aug 4. [Version 2] doi: 10.12688/wellcomeopenres.16901.2 (PMC9664024; doi:10.12688/wellcomeopenres.16901.2)
Supplement: Peer review history

# Peer review: Reviewer response for version 2

**DOI:** 10.21956/wellcomeopenres.20393.r53187

**Published:** 2022-11-14

**Authors:** Naiara Beraza, Meha Patel

**Affiliations:** 1 Gut Microbes and Health Institute Strategic Programme, Quadram Institute Bioscience, Norwich, UK

The authors have satisfactorily addressed the issues raised. We have no further comments to make.

Is the work clearly and accurately presented and does it cite the current literature?

Yes

If applicable, is the statistical analysis and its interpretation appropriate?

Yes

Are all the source data underlying the results available to ensure full reproducibility?

Yes

Is the study design appropriate and is the work technically sound?

Yes

Are the conclusions drawn adequately supported by the results?

Yes

Are sufficient details of methods and analysis provided to allow replication by others?

Partly

Reviewer Expertise:

Gastroenterology, immunology

We confirm that we have read this submission and believe that we have an appropriate level of expertise to confirm that it is of an acceptable scientific standard.

# Peer review: Reviewer response for version 1

**DOI:** 10.21956/wellcomeopenres.18645.r48286

**Published:** 2022-02-21

**Authors:** Gideon Hirschfield

**Affiliations:** 1 Toronto Centre for Liver Disease, University Health Network, University of Toronto, Toronto, ON, Canada

Nice study; important to try and extend the observations. The challenge for the field is getting studies that are adequately powered and which capture the clinical diversity of patients with PSC-UC and UC. The diseases are very variable, patients are on different treatments, and liver disease severity/recent infective complications are all hard to control. I didn't get the full sense of the clinical background for the participants. Furthermore the literature hasn't found a clear single answer to how the microbiome is involved in this disease. This paper is nevertheless well written. It is provocative and the findings need extension and validation but methodologically the data is well presented, and transparent. It sets the scene for larger collaborative efforts.

Is the work clearly and accurately presented and does it cite the current literature?

Yes

If applicable, is the statistical analysis and its interpretation appropriate?

Yes

Are all the source data underlying the results available to ensure full reproducibility?

Yes

Is the study design appropriate and is the work technically sound?

Yes

Are the conclusions drawn adequately supported by the results?

Partly

Are sufficient details of methods and analysis provided to allow replication by others?

Yes

Reviewer Expertise:

Cholestatic and autoimmune liver disease

I confirm that I have read this submission and believe that I have an appropriate level of expertise to confirm that it is of an acceptable scientific standard.

## References

[1] : Altered Gut Microbial Metabolism of Essential Nutrients in Primary Sclerosing Cholangitis. *Gastroenterology* .160(5) : 10.1053/j.gastro.2020.12.058 1784-1798.e0 10.1053/j.gastro.2020.12.058 33387530PMC7611822

# Response

**Published:** 2022-09-12

**Authors:** Nicholas Ilott

**Affiliations:** University of Oxford, UK

Reviewer #2: Professor Gideon Hirschfield

 Nice study; important to try and extend the observations. The challenge for the field is getting studies that are adequately powered and which capture the clinical diversity of patients with PSC-UC and UC. The diseases are very variable, patients are on different treatments, and liver disease severity/recent infective complications are all hard to control. I didn't get the full sense of the clinical background for the participants. Furthermore the literature hasn't found a clear single answer to how the microbiome is involved in this disease. This paper is nevertheless well written. It is provocative and the findings need extension and validation but methodologically the data is well presented, and transparent. It sets the scene for larger collaborative efforts.

**We would like to thank Professor Gideon Hirschfield for taking the time to review our manuscript. There are a number of challenges in the field and we agree with professor Hirschfield that this is a first step towards understanding molecular alterations in PSC-UC. Large, prospective cohorts with well curated metadata are indeed required to be able to disentangle the multiple host and/or microbial factors that contribute to both the development and the heterogeneity in PSC-UC.**

**We have included the reference provided by Professor Hirschfield in the introduction of the manuscript.**

# Peer review: Reviewer response for version 1

**DOI:** 10.21956/wellcomeopenres.18645.r48287

**Published:** 2022-02-15

**Authors:** Naiara Beraza, Meha Patel

**Affiliations:** 1 Gut Microbes and Health Institute Strategic Programme, Quadram Institute Bioscience, Norwich, UK

The article by Ilott NE *et al*. describes tissue-specific transcriptional analysis of different sites in the intestine in healthy individuals, PSC/UC and UC patients. In parallel, the authors show analysis of the mucosa-associated microbiome across the different intestinal tissue locations.

 The authors identify the reduction in *GGT1* expression in the ileum and cecum as the most relevant difference between PSC/UC and UC patients. The current study concludes that the changes in the mucosal microbiome are individual-dependent rather than tissue-dependent in the patients studied.

 There are some areas that should be addressed:

1. There is inconsistency in the number of participants detailed throughout the MS. For example, relating to RNAseq analyses, in the abstract it is said that there are n=8 PSC/UC, n=10 UC and n=12 healthy participants. In methods the number of samples collected for RNAseq are: n=9 PSC/UC, n=10 UC and n=13 healthy (page 3). Then in page 4, in “processing RNA-seq data” total numbers are: n=7 PSC/UC, n=10 UC and n=12 healthy.
2. Likewise, on methods it is said that n=16 control, n=20 PSC/UC and n=18 UC patients are included in the study for mucosal-microbiome analysis, while in Table 1 the numbers are different. Is the reason for this discrepancy that not all samples from the 3 different locations were obtained from the same patient? This is relevant to interpret the results, particularly when the authors conclude that differences in the microbiome are inter-individual but not tissue-location specific. The authors should clarify whether the different location-samples analysed were obtained from the same patients.
3. The population used seems heterogeneous in terms of medication use. For example, some UC patients were using azathioprine, which could influence the immune system/inflammation related results. Have the authors considered analysing the data by grouping patients by medication use or inflammation markers?
4. In Results, “Tissue-defining transcriptional…” (page 6) when describing Figure 1, the tissue-location of cluster 4 is not described but it is defined as enriched in adaptive immune responses (Fig 1C).
  Later in the text (page 7), when describing the enrichment of transcription factor binding motifs, cluster 4 is described as ‘mixed ileum-caecum’ (Fig 1K). The authors should clarify this.
5. Statistical significances should be included in Fig 2.
6. The authors should indicate if the mucosal samples analysed for bacterial composition were obtained from the same patients as the tissue samples analysed by RNAseq. This may be relevant to interpret the host-microbiome correlation analyses performed.

Is the work clearly and accurately presented and does it cite the current literature?

Yes

If applicable, is the statistical analysis and its interpretation appropriate?

Yes

Are all the source data underlying the results available to ensure full reproducibility?

Yes

Is the study design appropriate and is the work technically sound?

Yes

Are the conclusions drawn adequately supported by the results?

Yes

Are sufficient details of methods and analysis provided to allow replication by others?

Partly

Reviewer Expertise:

Gastroenterology, immunology

We confirm that we have read this submission and believe that we have an appropriate level of expertise to confirm that it is of an acceptable scientific standard, however we have significant reservations, as outlined above.

# Response

**Published:** 2022-09-14

**Authors:** Nicholas Ilott

**Affiliations:** University of Oxford, UK

The article by Ilott NE *et al*. describes tissue-specific transcriptional analysis of different sites in the intestine in healthy individuals, PSC/UC and UC patients. In parallel, the authors show analysis of the mucosa-associated microbiome across the different intestinal tissue locations.

 The authors identify the reduction in *GGT1* expression in the ileum and cecum as the most relevant difference between PSC/UC and UC patients. The current study concludes that the changes in the mucosal microbiome are individual-dependent rather than tissue-dependent in the patients studied.

 There are some areas that should be addressed:

 There is inconsistency in the number of participants detailed throughout the MS. For example, relating to RNAseq analyses, in the abstract it is said that there are n=8 PSC/UC, n=10 UC and n=12 healthy participants. In methods the number of samples collected for RNAseq are: n=9 PSC/UC, n=10 UC and n=13 healthy (page 3). Then in page 4, in “processing RNA-seq data” total numbers are: n=7 PSC/UC, n=10 UC and n=12 healthy.

**We would like to thank Dr Beraza and Dr Patel for taking the time to review our manuscript and providing constructive criticism.**

**Thank you for pointing out inconsistencies in patient/sample numbers. We agree that the numbers are confusing as currently used. The reason for the apparent discrepancies throughout the manuscript relate to the use of either patient numbers or sample numbers. While we endeavoured to capture all three tissue sites for each patient, samples that were eventually analysed i.e. ileum, caecum, rectum for these patients varies based on whether the sample was collected, processed and/or passed RNA-seq quality control. To address the issue and make it easier for the reader to discern the numbers, we have added the number of samples that were included in the final analysis for each tissue in each patient group in the abstract. We have left the methods section as it currently is as this reflects the number of unique patients recruited. Consistent with the changes in the abstract, we have altered the “processing RNA-seq data” section to reflect the total number of *samples *analysed per patient group. We hope that this clarifies the numbers.**

 Likewise, on methods it is said that n=16 control, n=20 PSC/UC and n=18 UC patients are included in the study for mucosal-microbiome analysis, while in Table 1 the numbers are different. Is the reason for this discrepancy that not all samples from the 3 different locations were obtained from the same patient? This is relevant to interpret the results, particularly when the authors conclude that differences in the microbiome are inter-individual but not tissue-location specific. The authors should clarify whether the different location-samples analysed were obtained from the same patients.

**As described for the RNA-seq data analysis above, we did attempt to capture all three tissue sites for each patient. However, samples that were eventually analysed i.e. ileum, caecum, rectum for these patients varies based on whether the sample was collected, processed and/or passed filtering post-16S rRNA amplicon sequence generation. As above, we prefer to leave the total number of unique patients that were recruited onto the study in the methods section. We have added a sentence describing that a cut-off of 5000 reads was used to filter out low quality samples (in the “Processing 16S rRNA amplicon sequencing” section). A reference to Table 1 is also included in this section to point to the breakdown of samples used in the analysis.**

 The population used seems heterogeneous in terms of medication use. For example, some UC patients were using azathioprine, which could influence the immune system/inflammation related results. Have the authors considered analysing the data by grouping patients by medication use or inflammation markers?

**The heterogeneity in the cohort in terms of medication use and/or inflammation status is noteworthy and we thank Dr Beraza and Dr Patel for bringing up this point. It is something that we considered and investigated in exploratory analyses and although these results are not presented in the final manuscript, details are available online at https://github.com/nickilott/PSC_UC_Host_Microbiome/tree/main/report. We also summarise the findings below (Figure 1). Firstly, we explored expression patterns of a range of pro-inflammatory marker genes across samples and in relation to variables of interest, including medication use. Although the majority of patients did not show signs of histological inflammation, we did observe substantial variation in expression of inflammatory markers. Indeed, these were more highly expressed in patients with PSC/UC and UC relative to healthy participants in the ileum (Figure 1 in this response, which can be viewed here). Nevertheless, there was not an obvious association with treatment for these markers. We therefore decided to continue the analysis without doing a sub-group analysis for either inflammation status or medication use. This is an important area of investigation that we feel cannot be reasonably addressed in this study due to relatively small sample size.**

 In Results, “Tissue-defining transcriptional…” (page 6) when describing Figure 1, the tissue-location of cluster 4 is not described but it is defined as enriched in adaptive immune responses (Fig 1C).

 Later in the text (page 7), when describing the enrichment of transcription factor binding motifs, cluster 4 is described as ‘mixed ileum-caecum’ (Fig 1K). The authors should clarify this.

**Thank you for spotting this omission. We have added this to the description of Figure 1 in the results section.**

 Statistical significances should be included in Fig 2.

**We have added these to Figure 2.**

 The authors should indicate if the mucosal samples analysed for bacterial composition were obtained from the same patients as the tissue samples analysed by RNAseq. This may be relevant to interpret the host-microbiome correlation analyses performed.

**A subset of the mucosal biopsies used for 16S rRNA amplicon sequencing had accompanying RNA-seq data. Indeed, RNA and DNA was extracted from this subset using dual DNA/RNA extraction kits enabling recovery of bacterial relative abundances and host transcription from the same piece of tissue. We apologise that this was not clear in the manuscript and have added a sentence in the methods (“Host-microbiome correlation analysis”) section to describe the number of RNAseq-16SrRNA pairs that were analysed in the correlation analyses.**
